# Static-gradient NMR imaging for depth-resolved molecular diffusion in amorphous regions in semicrystalline poly(tetrafluoroethylene) film

**DOI:** 10.5194/mr-6-317-2025

**Published:** 2025-12-15

**Authors:** Natsuki Kawabata, Naoki Asakawa, Teruo Kanki

**Affiliations:** 1 Material Science Program, Division of Materials and Environment, Graduate School of Science and Technology, Gunma University, Kiryu, Gunma 3768515, Japan; 2 Institute of Scientific and Industrial Research, Osaka University, Ibaraki, Osaka 567-0047, Japan

## Abstract

Understanding spatially heterogeneous molecular diffusion in semicrystalline polymers is critical for elucidating interfacial dynamics in soft materials. This study employs static-gradient nuclear magnetic resonance (NMR) imaging to capture the depth-resolved translational motion of polymer chains in a polytetrafluoroethylene (PTFE) film. By focusing on spin–spin relaxation behavior in amorphous regions near crystalline lamellae, we identify multiple diffusion regimes consistent with Bloch–Torrey analysis. The results reveal that molecular mobility at the substrate interface of PTFE film, immobilized on a glass substrate using epoxy resin, is significantly constrained, likely due to interfacial pinning, while the air-side surface shows signs of enhanced mobility. Our findings highlight the utility of static-gradient field NMR for probing nanoscale dynamical heterogeneity in semicrystalline systems.

## 1 Introduction

Heterogeneity in the dynamical behavior of polymer films – manifested as distinct molecular dynamics at the air-facing surface within the bulk and at the substrate interface – profoundly influences their physical properties, particularly dynamic viscoelasticity [Bibr bib1.bibx35]. Over the past few decades, considerable efforts have been devoted to clarifying how interfacial and surface regions contribute to phenomena such as the glass transition, highlighting the critical role of site-specific molecular mobility. To further unravel these complex dynamics, analytical techniques that can provide spatially resolved information about molecular motion in polymer thin films are indispensable.

Nuclear magnetic resonance (NMR) spectroscopy offers a powerful and noninvasive means of probing molecular structure and dynamics across a broad range of materials [Bibr bib1.bibx59]. Among NMR-based approaches, pulsed gradient spin-echo (PGSE) methods have been widely adopted because they enable both high-resolution spectroscopy and magnetic resonance imaging (MRI) [Bibr bib1.bibx8]. Nevertheless, while PGSE methods are well suited for liquid systems, the gradient strengths achievable in typical setups are often insufficient to study solid-state specimens or to capture diffusion processes with extremely small diffusion coefficients [Bibr bib1.bibx36]. These limitations underscore the need for alternative approaches that are specifically tailored to solid materials.

Furthermore, electrically conductive solid components within the sample can generate substantial eddy currents, potentially degrading the specimen. Because eddy currents distort the NMR signal, it is necessary to wait for an adequate period for their decay prior to acquiring a reliable measurement [Bibr bib1.bibx14]. In addition, conventional high-frequency NMR systems that rely on superconducting magnets demand extensive operational infrastructure – large-scale facilities, cryogenic cooling, and vacuum environments – which imposes significant financial and logistical burdens. Thus, there remains a pressing need for a simple, cost-effective, and versatile MRI method that can provide spatially resolved information on molecular dynamics in solid systems.

To overcome these challenges, methodologies utilizing static magnetic field gradients (SFGs) have been developed as compelling alternatives [Bibr bib1.bibx10]. While PGSE relies on pulsed gradients, SFG techniques use a continuously imposed gradient. Early implementations typically used fringe fields of electromagnets and were limited by thermal and power constraints, but later studies demonstrated that sufficiently strong gradients can be achieved using superconducting magnets [Bibr bib1.bibx36]. As a result, SFG-based approaches have enabled quantification of self-diffusion coefficients in solids and have inspired diverse, cost-effective platforms, including the superconducting fringe field (SFF) technique [Bibr bib1.bibx36], stray-field imaging (STRAFI) using GARField magnets [Bibr bib1.bibx18], anti-Helmholtz superconducting magnets [Bibr bib1.bibx10], NMR MOUSE [Bibr bib1.bibx22], single-sided NMR systems [Bibr bib1.bibx7], bulk high-temperature superconducting magnet-based systems [Bibr bib1.bibx54], Halbach-array NMR sensors [Bibr bib1.bibx50], and compact ferromagnet-based MRI systems [Bibr bib1.bibx2]. These developments clearly demonstrate the potential of SFG-based MRI to complement or even replace conventional PGSE approaches, especially for solid materials.

However, another complication arises from the fact that, in many SFG-based NMR systems, the static magnetic field governing resonance conditions and the magnetic field gradient used for imaging or diffusion measurements cannot be independently controlled. This interdependence complicates NMR measurements across different frequencies while maintaining consistent spatial resolution. Nevertheless, spatially resolved measurements of spectral density functions – i.e., local spectral densities – are increasingly in demand, as they enable advanced imaging modalities. Addressing these challenges calls for the development of novel SFG-based MRI methodologies that combine simplicity, tunability, and sensitivity to solid-state molecular motion.

In this context, the present study introduces a novel, nondestructive, and facile molecular dynamics imaging technique. This approach employs a locally generated magnetic field gradient produced from a needle-shaped ferromagnetic material developed in-house. Using this technique, we perform depth-resolved spin–spin relaxation rate ( $R_{2}$ ) imaging of a polymer film, enabling direct comparison of molecular dynamics at the film surface and near the substrate interface.

In our previous study, we measured variable-frequency spin–lattice relaxation rates ( $R_{1}$ ) to determine the spectral density function associated with spatially resolved local molecular motion [Bibr bib1.bibx33]. Although the thicknesses of the surface and interfacial regions estimated from $R_{1}$ variations were overestimated due to ^19^F–^19^F spin diffusion, a clear disparity between the surface/interface and interior of the PTFE film was confirmed. However, the $R_{1}$ values showed no discernible differences between the air-side surface and the polymer–substrate interface. This result contrasts with the widely reported behavior of conventional glass-forming polymer thin films, where $T_{g}$ typically decreases at the free surface [Bibr bib1.bibx24] and increases near the substrate interface [Bibr bib1.bibx40]. This discrepancy may arise because $R_{1}$ reflects only rotational (more precisely, reorientational) molecular dynamics and is insensitive to translational diffusion.

To complement our previous work, we examined the influence of translational diffusion on spin–spin relaxation using an $R_{2}$ dispersion approach [Bibr bib1.bibx60] by systematically varying the echo time in the Carr–Purcell–Meiboom–Gill (CPMG) sequence [Bibr bib1.bibx9]. The CPMG sequence was employed for the MRI measurements, and the dependence of $R_{2}$ on translational diffusion was examined by varying the half echo time, τ , as follows: depth-resolved one-dimensional imaging was achieved by stepwise modulation of the static magnetic field strength using a normal-conducting electromagnet. The RF intensity for the CPMG method was nominal 50 kHz, which was calibrated using a CPMG pulse sequence under a homogeneous resonant magnetic field only from the electromagnet. At each magnetic field point, 256 signal accumulations were acquired at a resonance frequency of 29.750000 MHz. The decay plots and fitting curves for the CPMG measurements are shown in Appendix A. Furthermore, the influence of the intrinsic $R_{2}$ on the experimentally obtained $R_{2}$ was negligibly small (see Appendix B). However, in the $R_{2}$ dispersion method employed in this study, accurately determining the diffusion coefficient is challenging due to the ambiguous nature of the local magnetic field gradient within the sample. Consequently, it should be emphasized that the diffusion analysis presented here is qualitative in nature. It should be noted that the PTFE sample studied here is a highly crystalline polymer, with a degree of crystallinity of approximately 90 % [Bibr bib1.bibx33]. In the CPMG spin-echo method used in this study, NMR signals from the crystalline regions are expected to decay rapidly due to strong ^19^F–^19^F dipolar interactions, and thus they do not contribute significantly to the observed spin echoes. The detected NMR signals primarily arise from amorphous regions located at the surfaces of crystalline grains. Although these amorphous molecules are in a rubbery state, their diffusion is restricted by surrounding molecules and crystalline domains. Therefore, as discussed later, the diffusion characterized in this study represents motion within compartmentalized and confined amorphous spaces.

## 2 Methods

### 2.1 Principle of depth profiling

The magnetic field gradient experienced by the sample arises from the combined influence of the static magnetic field and its gradient along the thickness of the polymer film. This gradient is induced by a needle-shaped ferromagnetic material and the uniform external static magnetic field generated by the electromagnet. In our one-dimensional MRI approach, we fix the resonance frequency and apply a spatially selective RF pulse with an RF field strength of nominal 50 kHz. This pulse excites nuclear spins only within a narrow slice (the NMR-active slice; NAS), whose spatial position is defined by the static field gradient at the chosen resonance frequency. By incrementally varying the static magnetic field, the effective field gradient experienced by the sample changes, and consequently, the position of the NMR-active slice is shifted along the thickness direction of the film (Fig. [Fig F1]). This procedure enables one-dimensional imaging along the sample depth without the need for frequency-swept selective excitation or gradient switching during RF irradiation. A similar principle – distance encoding realized by the translation of a spatially selective sensitive volume – has long been employed in well-logging NMR [Bibr bib1.bibx30]. Our method shares the same fundamental mechanism: the imaging contrast arises from the interplay between a localized excitation region and its systematic displacement through the sample. This conceptual parallel underscores the validity of our approach and places it within the broader class of one-dimensional imaging methods that rely on spatial selectivity and controlled volume movement.

Within this framework, the spin density or molecular dynamics at a specific location within the sample can be characterized by analyzing the NMR signal arising from the intersection volume between the excited volume and the sample. The morphology of the excited volume, which is shaped by the presence of a needle-shaped ferromagnetic material, is anticipated to adopt a concave geometry, as illustrated in Fig. [Fig F1]a [Bibr bib1.bibx17]. The excited volume can be displaced vertically by modulating the strength of the external static magnetic field produced by the electromagnet, as shown in Fig. [Fig F1]a and b. Systematic variation in the external static magnetic field enabled the acquisition of a depth-resolved profile of the sample.

**Figure 1 F1:**
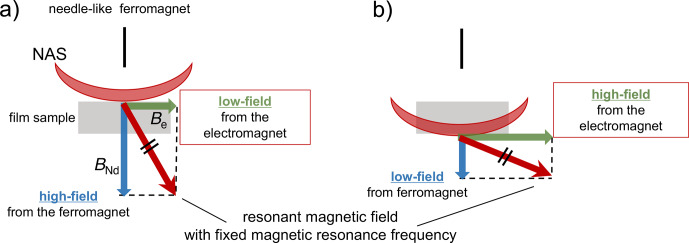
In our one-dimensional MRI approach, the resonance frequency was fixed, and a spatially selective RF pulse with an RF field strength of nominal 50 kHz was applied. This pulse excites nuclear spins only within a narrow slice (the NMR-active slice; NAS), whose spatial position is defined by the static field gradient at the chosen resonance frequency. By incrementally varying the static magnetic field, the effective field gradient experienced by the sample changes, and consequently, the position of the NAS is shifted along the thickness direction of the film. The combined effect of the magnetic field generated by the needlelike ferromagnet, $B_{\text{Nd}}$ , and the static magnetic field from the electromagnet, $B_{e}$ , gives rise to the mechanism of movement of the NMR-active slice.

### 2.2 Experimental setup

The experimental setup for this methodology is illustrated in Fig. [Fig F2]. A spherical neodymium magnet (8 mm diameter sourced from TRUSCO Nakayama Corporation) and an iron needle (1 mm diameter with a tip diameter of 0.2 mm) were affixed to the aluminum jig. By bringing them in direct contact, the iron needle is magnetized and transformed into a ferromagnetic needle that functions as a localized ferromagnetic material. The needle was positioned between the poles of a water-cooled electromagnet, serving simultaneously as a static magnetic field source and static magnetic field gradient generator. For detailed specifications of the apparatus, refer to our previously published work [Bibr bib1.bibx32].

**Figure 2 F2:**
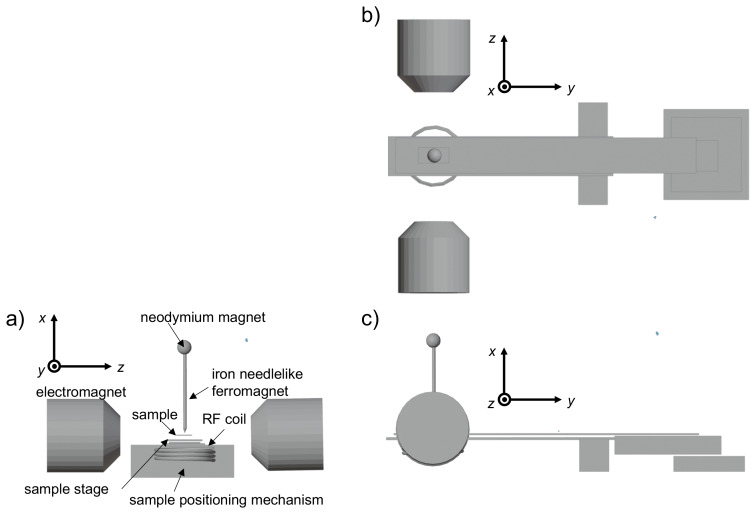
Schematic illustration of an MRI probe. Three-dimensional views from the y axis **(a)**, x axis **(b)**, and z axis **(c)** are shown. A small spherical neodymium magnet magnetizes the paramagnetic needle, converting it into a needlelike ferromagnet. A two-dimensional mechanical scanning stage is shown (only the function for sample positioning is used in this work, not the scanning function).

## 3 Results and discussion

### 3.1 Depth-direction one-dimensional spin–spin relaxation rate imaging of a single layer of polymer film

To investigate the effect of translational molecular diffusion at the film surface and the polymer–glass interface, we performed one-dimensional imaging of the spin–spin relaxation rate ( $R_{2}$ ) of ^19^F nuclei along the depth of a 2 mm thick PTFE film immobilized on a glass substrate using epoxy resin. From the blurred image of the sample, which is determined from magnetization intensity, this method achieves spatial resolution of the order of sub-millimeter scale, which enables observation of mesoscopic heterogeneities.

The resulting $R_{2}$ imaging data are presented in Fig. [Fig F3], where $M_{\inf}$ represents the NMR signal intensity. In this imaging approach, the horizontal axis corresponds to the static magnetic field strength generated by the electromagnet, with an increasing field strength corresponding to deeper regions within the sample. Thus, the region of lower static magnetic field strength represents the air-facing surface of the film, whereas the region of higher static magnetic field strength corresponds to the interface with the glass substrate. As shown in Fig. [Fig F3], no variations in $R_{2}$ are present near the air-side surface of the film ( $B_{e}<0.762T$ ) for different τ values. A similar trend was observed in the interior of the film. However, near the glass-side interface of the film ( $B_{e}>0.766T$ ), complex behavior in $R_{2}$ emerged. Specifically, as τ increases, $R_{2}$ initially exhibits an increase ( τ=30 µs ) before subsequently decreasing for longer τ values ( τ=40 µs and τ=50 µs ). The contrasting behavior of $R_{2}$ at the air- and glass-side interfaces of the film could be attributed to different molecular interactions. At the air interface, PTFE molecules behave as free ends owing to the surface energy effects of interactions with adjacent PTFE molecules. Conversely, near the glass substrate interface via epoxy resin, the pinning effect induced by interactions between epoxy resin and PTFE molecules is presumed to constrain translational diffusion, thereby influencing the observed relaxation dynamics. 

**Figure 3 F3:**
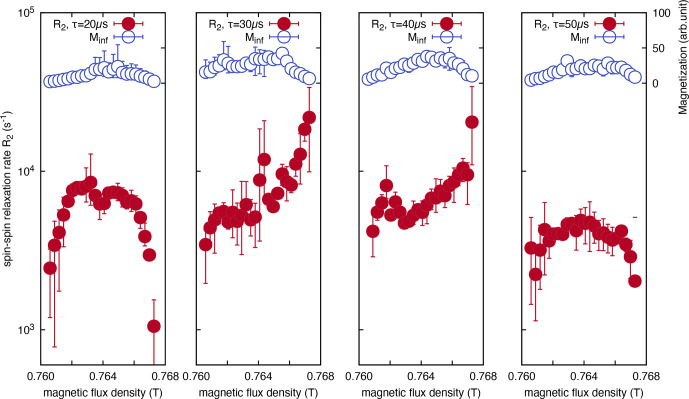
Magnetic field dependence (corresponding to depth dependence) of the $T_{2}$ relaxation rate ( $R_{2}$ ), measured using the CPMG method at different echo times for a PTFE film, adhered to a soda-lime glass substrate with epoxy resin. $R_{2}$ rate showed minimal contrast at the PTFE film surface ( $B_{e}<0.762T$ ), whereas it displayed remarkable changes at the substrate interface ( $B_{e}>0.766T$ ).

Based on the experimental findings presented above, we hypothesize that the variation in the dependence of $R_{2}$ on increasing τ arises from differences in the translational diffusion effects. This is modulated by the distinct interfacial environments of the PTFE film, such as air or the glass substrate.

### 3.2 Three diffusional regimes and regime transitions

Figure [Fig F4]a illustrates the results of numerical simulations using the Bloch–Torrey equation (see Appendix C). The variation in diffusion regime with the dimensionless diffusion coefficient $\tilde{D}(=D\tau /L_{s}^{2}$ ) and position x of the nuclear spin in real space is represented on the xy axes of the plot. The contribution of the relaxation exponent of the spin–spin relaxation rate $R_{2}$ due to diffusion is depicted along the z axis. When the echo time τ is short, the spins have not yet reached the diffusion barrier and therefore undergo free diffusion. Therefore, spins remain within the short-time regime. Here, the molecule is free to diffuse throughout the space. As molecular diffusion advances and the molecule approaches the diffusion barrier, it transitions into a localization regime where diffusion is constrained by the barrier. As the diffusion continues, the molecule enters the motional averaging regime, undergoing multiple round trips between the diffusion barriers.

In this regime, molecular motion undergoes averaging such that the system appears stationary and diffusion is not observed. Given the difficulty of observing the three-dimensional curve in Fig. [Fig F4]a, the sum of the magnetization versus position in real space is plotted with respect to $\tilde{D}$ in Fig. [Fig F4]b. The figure shows the presence of three distinct regimes.

**Figure 4 F4:**
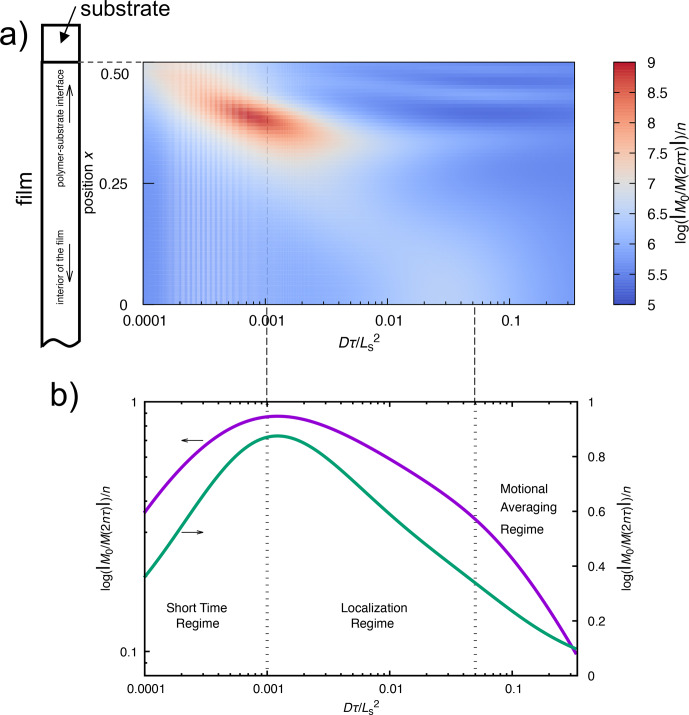
Simulation of the relaxation exponent of the first echo signal of the CPMG experiment using the Bloch–Torrey equation for the diffusion of nuclear spins in a compartmentalized environment. The contribution of diffusion to the spin–spin relaxation rate $R_{2}$ is color-coded **(a)**, where the relaxation exponent of the CPMG echo intensity M(2nτ) (for n=1 ) is plotted as a function of the dimensionless diffusion coefficient (or dimensionless echo time) and position within the sample. The position is defined relative to the midpoint between diffusion barriers of the compartment, with a magnetic field gradient symmetrically distributed about the origin, having the form $g(x)=x^{4}$ . The figure presents data for only half of the compartment. The simulation models a polymer film confined between two substrates in order to apply periodic boundary conditions. In practice, however, only one side of the polymer film adheres to the substrate, while the opposite surface is exposed to air. Thus, the film possesses an asymmetric structure along the depth direction, with one interface in contact with the substrate and the other with air. Consequently, only the results corresponding to the substrate–film interface are displayed. The dimensionless diffusion coefficient is expressed as $D\tau /L_{s}^{2}$ , where $L_{s}$ represents the distance between the diffusion barriers of the compartment, D is the diffusion coefficient, and τ is the CPMG echo time. Panel **(b)** illustrates the total observed magnetization within the compartment as a function of the dimensionless diffusion coefficient, highlighting the three regimes.

### 3.3 Distinction between surface and interface

Building on the aforementioned observations, we examine the behavior of $R_{2}$ . A consistent trend was observed across all echo times. τ conditions used in the experiment were as follows: $R_{2}$ was notably smaller on the air-side surface of the PTFE film ( $B_{e}<0.762T$ ) than in the interior of the film. The observed difference in $R_{2}$ between the surface and the interior of the film originates from intrinsic variations in the local polymer morphology rather than from differences in diffusion. A reduction in $R_{2}$ on the air-side surface was observed irrespective of the variation in τ . Results from variable-frequency $R_{1}$ measurements [Bibr bib1.bibx33] indicated that, within the resonant frequency range (29.75 MHz) employed for the $R_{2}$ measurements, the $R_{1}$ value near the surface was 1.5–2 orders of magnitude larger than the $R_{1}$ value of the film. Hence, the contributions of $R_{1}$ to $R_{2}$ , that is, the effect of the secular term, were considered negligible. Therefore, we hypothesize that a reduction in $R_{2}$ observed at the film surface can be attributed to differences in the zero-frequency component of the spectral density function, J(ω=0) . Based on the established variation in the spectral density of molecular motion near the film surface or substrate interface [Bibr bib1.bibx33], PTFE molecules are postulated to exhibit enhanced molecular motion, particularly the reorientational motion at zero or low frequencies below several hundred kilohertz, compared to the bulk of the film. It should again be emphasized that the observed thicknesses of the surface and interfacial regions are significantly greater than the typical values – of the order of several tens of nanometers – commonly reported for surfaces and interfaces in nanometer-scale thin films [Bibr bib1.bibx24]. This discrepancy is attributable to the spin diffusion effect of ^19^F–^19^F interactions on $R_{2}$ .

#### 3.3.1 Behavior of the air surface and interior of the polymeric film

We now examine the impact of variations in the echo time τ on the air-side surface and the interior of the PTFE film. The results demonstrate that the value of $R_{2}$ remains largely unchanged even when the echo time τ gradually increases. By comparing this behavior with Fig. [Fig F4]a, the system is in the localization regime, where changes in τ exert a minimal influence on $R_{2}$ . This suggests that the air-side surface acts as a free end, where the dynamics of individual polymer chains are not entirely random, and that these chains function as a diffusion barrier in the direction normal to the film surface because of their interactions with adjacent polymer chains. Moreover, since the $R_{2}$ value of the PTFE molecules within the film was independent of the variations in τ , the behavior observed on the film surface can be attributed to the film interior. However, within the film, unlike the one-dimensional diffusion barrier perpendicular to the film surface, localization is presumed to arise from collisions with the three-dimensional barriers formed by the surrounding PTFE molecules.

#### 3.3.2 Behavior of the interface between the polymeric film and the substrate

We now focus on the region near the interface between the PTFE film and the glass substrate via epoxy resin. Near the glass-side interface of the PTFE film ( $B_{e}>0.766T$ ), $R_{2}$ exhibits notable variation when τ shifts from 20 to 30 µs . However, as τ reaches 40 µs , the value of $R_{2}$ begins to decline, and by the time τ reaches 50 µs , $R_{2}$ decreases precipitously. The observed behavior can be attributed to a transition of the observable from the localization regime to the averaging regime with increasing τ .

### 3.4 Pinning effect at interface

The diffusion behavior of PTFE molecules near the PTFE film interface differed from that in the film interior or near the film surface, owing to the influence of the epoxy resin. This disparity is due to employing the same sample for all the experiments conducted at 298 K, under the assumption that the diffusion coefficient D remains constant under isothermal conditions. Here, D represents the diffusion coefficient as the statistical average of random motion, akin to Brownian motion. In other words, D corresponds to the diffusion coefficient in the Fokker–Planck equation, which is used when the Langevin equation for a single molecule that accounts for random forces due to thermal fluctuations at a given temperature is extended to a molecular ensemble. In the case that D and τ are constants, in $\tilde{D}$ (= $D\tau /L_{g}^{2}$ ), only $L_{g}$ denotes a variable. Here, the distance between diffusion barriers $L_{s}$ is replaced by the effective diffusion barrier distance, which is denoted as the spin packet length $L_{g}$ (see Appendix D for more details).

Specifically, when τ was set to 20 µs , a regime transition was observed in the depth direction of the polymer film, as depicted in the plot for τ=20 µs in Fig. [Fig F3]. This transition occurred between the localization and motional averaging regimes. Since D and τ are constants and the variations in $\tilde{D}$ are attributed to the changes in $L_{g}$ , the transition is triggered by a reduction in the spin packet length $L_{g}$ .

Near the substrate interface, significant changes in τ cause $R_{2}$ to transition from increasing to decreasing. Thus, a reduction in $L_{g}$ and an increase in τ lead to an increase in $\tilde{D}$ , which results in regime transition. Here, we explored this phenomenon. On the air-side surface of the PTFE film, despite the variations in τ during CPMG measurements, the value of $R_{2}$ exhibited a minimal change, indicating that the molecules on the air-side surface of the film were in the localization regime. In other words, the PTFE molecules on the air-side surface exhibited behavior consistent with restricted diffusion. However, when considered in conjunction with the experimental results for $R_{1}$ reported previously [Bibr bib1.bibx33], the effect of translational diffusion was found to be equivalent to that observed on the air-side surface and in the interior of the PTFE film, with the observed difference in $R_{2}$ arising from variations in the spectral density function, J(ω=0) , which is attributed to reorientational motion. This conclusion differs from the well-known results of translational diffusion of glassy polymers near the air-side surface, as revealed by fluorescence lifetime experiments and coarse-grained molecular dynamics simulations [Bibr bib1.bibx56], in which a polymeric thin film shows a decrease in the glass transition temperature at the surface. This discrepancy may be attributed to the fact that our $R_{2}$ dispersion experiments were carried out under rubbery-state conditions, at temperatures significantly higher than $T_{g}$ .

On the other hand, the observed relationship for the glass–substrate interface can be explained as follows: the spin packet length $L_{g}$ diminishes near the glass–substrate interface compared with that within the bulk of the film. There are two potential explanations for the reduction in $L_{g}$ . The first explanation involves an increase in the strength of the local magnetic field gradient owing to the contrast in magnetic susceptibility at the interface between the PTFE film and epoxy resin on the glass substrate. However, in this case, the effect of diffusion on $R_{2}$ increases monotonically. This is because when the magnetic field gradient strength is simply enhanced, the transition point between the localization regime and the motional averaging regime shifts to a larger $\tilde{D}$ , making it challenging to traverse the transition point by merely increasing τ . In this study, the transition from an increase to a decrease in $R_{2}$ owing to an increase in τ , that is, a regime transition, was observed, suggesting that the contribution from changes in the local magnetic field gradient is relatively minor compared with the contribution from the increase in τ . That is, a reduction in $L_{g}$ can be attributed to the second potential explanation, the diffusion of PTFE molecules. We speculate that the most likely mechanism for this phenomenon is the pinning effect of PTFE chains at the interface between the PTFE film and glass substrate. This pinning effect led to a reduction in $L_{s}$ , which subsequently results in a decrease in $L_{g}$ .

To provide a unified interpretation of this phenomenon, we applied the Bloch–Torrey equation, which integrates the effects of diffusion into the Bloch equation and the CPMG spin-echo experiment, followed by analyzing the diffusion regime transition in one-dimensional MRI. As illustrated in Fig. [Fig F4]a), compared with the interior of the film, which is distant from the diffusion barrier, the regime transition occurs at a smaller $\tilde{D}$ near the diffusion barrier where the magnetic field gradient is substantial.

## 4 Conclusions

As described above, NMR $R_{2}$ imaging of the depth profile of a polymer film, utilizing a static magnetic field gradient generated by a needle-shaped ferromagnetic material, revealed distinct variations in diffusion behavior near the air-side surface of a PTFE polymer film compared to the film interior and substrate-side interface. The methodology developed in this study offers a novel approach for imaging heterogeneous materials and dynamic imaging of molecular processes.

## Figures and Tables

**Figure A1:**
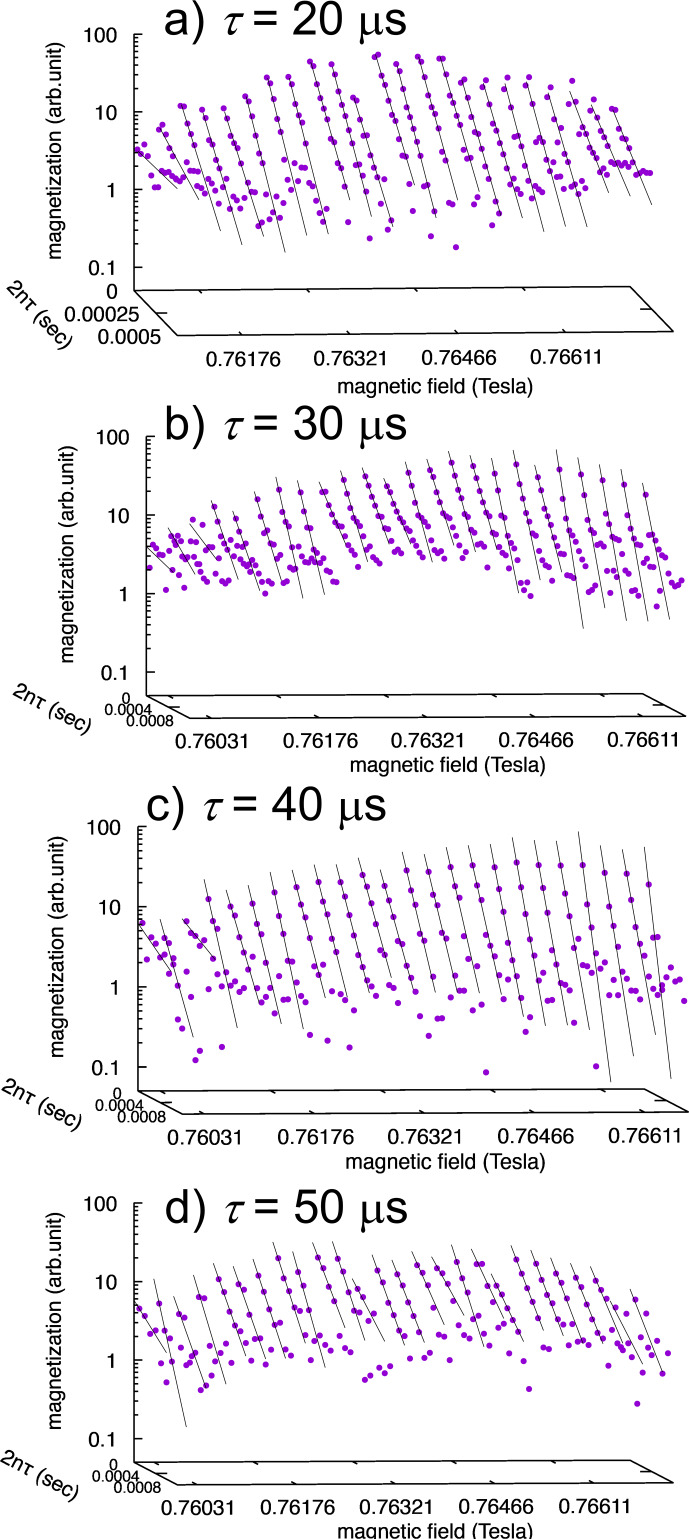
One-dimensional depth profiling of the CPMG decays for the PTFE film. The decay was fit using $M(2n\tau )=M_{\text{inf}}\mathrm{exp}(-R_{2}\tau )$ for the early time period of each decay.

## Data Availability

The simulation codes as well as datasets generated using the code for this study can be found on Zenodo (10.5281/zenodo.17893540; [Bibr bib1.bibx34]).

## Appendix A $R_{2}$ analyses from CPMG measurements

For CPMG measurements, the plots of decay data for each excited volume as a function of 2nτ are shown in Fig. [Fig F1]. Here, we discuss the determination of $R_{2}$ . The one-dimensional images reconstructed from the acquired signal intensities appear as blurred images, rather than reflecting the true rectangular geometry of the sample, resulting in broadened profiles. These blurred images arise from the convolution of the true sample geometry with the point spread function, which itself is determined by the spatial dependence of the magnetic field strength within the sample. Consequently, the data points in adjacent regions of the image do not originate from spatially independent excited volumes but instead represent correlated points. While this characteristic constitutes a limitation of the present method, it also suggests the possibility that, in the future, inverse problem approaches to convolution – such as the Landweber iteration method [Bibr bib1.bibx17] – may enable imaging with significantly enhanced spatial resolution.

## Appendix B Intrinsic $R_{2}$

Distinguishing between the intrinsic $R_{2}$ and the contribution arising from diffusion is crucial for accurately evaluating the influence of diffusion on $R_{2}$ . However, when the intrinsic $R_{2}$ lies within the short-time regime, namely the free-diffusion regime, it can be determined by extrapolating the $R_{2}$ vs. τ plot to τ→0 . Similarly, when the diffusion process resides in the motional averaging regime, the intrinsic $R_{2}$ can also be estimated. In contrast to the short-time regime, however, extrapolation concerning τ is not possible; instead, if the plot exhibits a value asymptotically approached with increasing τ , this value can be regarded as the intrinsic $R_{2}$ . By contrast, when the system falls into the localization regime, these methods cannot be applied. Furthermore, as demonstrated in the present study, in cases where regime transitions occur with variations in τ , determination of the intrinsic $R_{2}$ may not be straightforward. Therefore, we attempted to estimate the intrinsic $R_{2}$ from the τ dependence of $R_{2}$ in the motional averaging regime. However, since the τ dependence did not exhibit a clear asymptotic value, the intrinsic $R_{2}$ could not be determined. Nevertheless, from the peak-like feature observed in the τ dependence of $R_{2}$ near the interface with the substrate, it can be inferred that $R_{2}$ already decreases to approximately $10^{2}$ s^−1^ or less at an echo time of τ=100 µs . This suggests that the maximum intrinsic $R_{2}$ is of the order of $10^{2}$ s^−1^ and is likely smaller.

According to the Solomon–Bloembergen theory [Bibr bib1.bibx52], using a correlation time of 1 µs estimated from $R_{1}$ and the second moment of the ^19^F–^19^F magnetic dipolar coupling, $M_{2}=(2\pi \times 36.568$ kHz)^2^ = 5.27 × 10^10^ s^−2^, the relaxation rate can be estimated as $R_{2}\approx M_{2}\tau_{c}\approx 1\times 10^{4}$ s^−1^. This value exceeds the experimentally obtained $R_{2}$ , indicating that employing the correlation time corresponding to crystalline domains is not appropriate. In the CPMG method, signals from crystalline regions are attenuated by rapid $R_{2}$ relaxation during the echo intervals; therefore, the observed signals in this study are considered to originate solely from the amorphous regions. By adopting the reported correlation time of $\tau_{c}=4.3\times 10^{-14}$ s for the amorphous phase, $R_{2}$ is estimated as $2.3\times 10^{-3}$ s^−1^. Assuming that the intrinsic $R_{2}$ contributes within the range of $10^{-3}$ – $10^{2}$ s^−1^ to the experimentally observed $R_{2}$ , this contribution is negligibly small. Consequently, in this work, the experimentally obtained $R_{2}$ values were plotted without subtracting the intrinsic $R_{2}$ component.

## Appendix C Numerical simulation

Magnetization decay in a spin-echo experiment subjected to a static magnetic field gradient can be simulated by numerically solving the Bloch–Torrey equation [Bibr bib1.bibx58]. This section presents a solution to the Bloch–Torrey equation within a compartmentalized diffusion environment. In this context, the compartments emulate the cage effect imposed by the surrounding molecular segments of the polymer chains, a phenomenon frequently considered in the study of glass transition behavior [Bibr bib1.bibx20] or amorphous polymer chains between crystalline regions. Although the surface morphology of semicrystalline PTFE is not yet fully established, a study on AFM has reported a multiscale hierarchical surface structure [Bibr bib1.bibx37]. At the ∼100 µm scale, large domains ( ∼20 µm ) interconnected by rope-like features are observed. At higher spatial resolution ( ∼100 nm), highly oriented crystalline regions, presumably lamellae of the order of ∼100 nm, are separated by amorphous regions of several tens of nanometers. In our study, the characteristic distance between diffusion barriers, $L_{s}$ , is therefore presumed to be of the order of several tens of nanometers. However, our technique does not allow direct determination of $L_{s}$ or the effective spin packet length $L_{g}$ because the effective magnetic field gradient contains a significant but unknown local component arising from the intrinsic magnetic-susceptibility variations in the PTFE sample.

The Bloch–Torrey equation is given by

C1
$$\frac{\partial M}{\partial t}=\tilde{D}\frac{\partial^{2}M}{\partial {\tilde{x}}^{2}}-i\tilde{\gamma }G(\tilde{x})M,$$

where the tilde denotes the dimensionless parameters, allowing the problem to be treated in a generalized framework. The contributions of the longitudinal relaxation ( $R_{1}$ ) and intrinsic transverse relaxation ( $R_{2}$ ) in the original Bloch–Torrey equation were neglected in this calculation. Although these effects must be accounted for in practical applications, it is found that the effects are negligible in our case.

The dimensionless diffusion coefficient $\tilde{D}$ , the dimensionless gyromagnetic ratio $\tilde{\gamma }$ , and the dimensionless one-dimensional coordinates $\tilde{x}$ are defined as

C2D~=DτLs,C3γ~=τgγLs,C4x~=xLs,

where D , $L_{s}$ , γ , and x represent the diffusion coefficient, the characteristic length of the aforementioned compartment, the gyromagnetic ratio of a noticed nuclear spin, and the spatial position of the nucleus within the molecule, respectively.

Because the temporal sequence during echo time τ is irrelevant in transverse relaxation measurements, τ can be considered to be a single computational step. The time evolution operator for the spin-echo experiment over echo time τ is given by

C5
$$U_{+}={\text{e}}^{(\hat{W}-i\tilde{\gamma }\hat{B})\tau }.$$

Thus, the propagator of a two-pulse Hahn echo [Bibr bib1.bibx29] can be expressed as follows:

C6
$$U_{2\tau }=U_{-}U_{+}=(U_{+}{)}^{*}U_{+}.$$

Similarly, the propagator of the second echo in the CPMG sequence is given by

C7
$$U_{4\tau }=U_{+}U_{-}U_{-}U_{+}.$$

Extending this to the n th echo in the CPMG sequence, that is, the propagator, gives

C8
$$U_{2n\tau }=U_{2\tau }^{\mathrm{mod}(n,2)}U_{4\tau }^{\mathrm{floor}(n,2)}.$$

Consequently, the k th Fourier component of the magnetization at time t=2nτ is computed as follows:

C9
$$M(k,t)=U_{2n\tau }M(k,0).$$

Initial magnetization M(k,0) is determined using the following initial conditions $\left(\phi (\tilde{x},0\right)$ ):

C10∑k=0∞M(k,0)ψk(x~)C11=∑k=0∞M(k,0)cos⁡πkx~+12C12=ϕ(x~).

The Fourier component M(k,0) of the initial magnetization can be obtained using the inverse Fourier transform of $\phi (\tilde{x})$ . For instance, for a homogeneous spin density, where $\phi (\tilde{x})=1$ (constant), all the Fourier components vanish, except for k=0 because the inverse Fourier transform yields a delta function. Substituting the obtained M(k,0) into Eq. ([Disp-formula App1.Ch1.S3.E9]), the k th magnetization, M(k,t) at time t(=2nτ ), can be determined. Finally, the magnetization in real space M(x,t) is obtained using the inverse spatial Fourier transform of M(k,t) :

C13
$$M(x,t)=F^{-1}[M(k,t)].$$

In panel (a) of Fig. 4 in the main text, it is not straightforward to clearly identify the three diffusion regimes; therefore, in panel (b), we presented the vertical axis as the spatial summation of magnetization over all positions x . By stating that the results were “mapped to real space”, we meant that, after solving the Bloch–Torrey equation in Fourier space, we applied the inverse Fourier transform to recover M(x,t) in real space. The plot in panel (b) of Fig. 4 in the main text further shows $\sum_{x}M(x,t)$ as a function of the dimensionless echo time, representing the total magnetization integrated over all positions. The purpose of panel (b) was thus to make the visualization of the three diffusion regimes more accessible. Panel (a), on the other hand, demonstrates that in one-dimensional imaging, the echo times at which regime transitions occur differ between regions near the substrate interface and those deeper within the film.

A Fourier transform approach was employed to solve the Bloch–Torrey equation, which implicitly imposes periodic boundary conditions. In the case of a PTFE film sandwiched between two substrates, the interactions at both film surfaces with the substrates create a symmetric structure along the film thickness, making the direct application of periodic boundary conditions appropriate. However, in the present study, the polymer film exhibits an asymmetric structure, with one surface exposed to air and the other interfacing with the substrate. Therefore, we used only the simulation results corresponding to the half-depth of the compartment adjacent to the substrate, as shown in Fig. 4a in the main text.

## Appendix D Spin packet length

Here, we elucidate the concept of spin packet length, $L_{g}$ [Bibr bib1.bibx39]. The spin packet length represents the characteristic length scale associated with molecular diffusion. As mentioned previously, the distance between the diffusion barriers, which defines the spatial range over which molecules can diffuse, is denoted by $L_{s}$ . In NMR measurements, $L_{s}$ refers to the spatial extent to which PTFE molecules can move. However, in practical NMR relaxation measurements, the spatial region contributing to the magnetization is restricted, and not all nuclear spins within the sample are detected by NMR. In other words, in regions exposed to a strong magnetic field gradient, where magnetization relaxation is completed during the echo time before the signal is observed, such as near the diffusion barrier, the magnetization remains undetected even in regions where spins are present [Bibr bib1.bibx39].

Furthermore, even when considering a specific NAS in an MRI experiment, detecting NMR signals from all nuclear spins of interest within the NAS is impossible. Therefore, the effective diffusion barrier distance may be smaller than the physical distance. The spin packet length $L_{g}$ is the spatial region in which the NMR-active spins can be detected [Bibr bib1.bibx61]. Theoretical studies have indicated that this region, where spins are present but difficult to measure, is influenced by variations in the magnetic field gradient experienced by the sample [Bibr bib1.bibx39]. If the diffusion distance of the molecules is measured under varying magnetic field gradient strengths, the spin packet length $L_{g}$ may vary. However, in the experiment, the distance d , a parameter of the magnetic field gradient strength, was held constant between the tip of the needlelike ferromagnetic material and the sample surface, ensuring that the magnetic field gradient remained stable over time. Therefore, as a first approximation, the distance $L_{s}$ between the diffusion barriers and the observed spin packet length $L_{g}$ can be considered to be proportional. Consequently, for the interpretation of $R_{2}$ , we choose to use the spin packet length $L_{g}$ instead of $L_{s}$ .
